# Feasibility and safety of 1-min sit-to-stand test in acute decompensated heart failure confirmed by lung ultrasound

**DOI:** 10.3389/fcvm.2023.1103247

**Published:** 2023-03-08

**Authors:** Xiu Zhang, Yu Kang, Zeruxin Luo, Qiaowei Chen, Mengxuan Yang, Jijuan Zeng, Pengming Yu, Qing Zhang

**Affiliations:** ^1^Department of Rehabilitation Medicine Center, West China Hospital, Sichuan University, Chengdu, China; ^2^Department of Cardiology, West China Hospital, Sichuan University, Chengdu, China

**Keywords:** acute decompensated heart failure, 1-min sit-to-stand test, safety, lung ultrasound, feasibility

## Abstract

**Aim:**

This study innovatively proposed the 1-min sit-to-stand test (1-min STST) as an assessment tool for functional capacity in acute decompensated heart failure (ADHF), in which its feasibility and safety were investigated.

**Methods:**

This was a prospective, single-center cohort study. The 1-min STST was performed after the first 48 h of admission when vital signs and Borg score were collected. Lung ultrasound was used to measure pulmonary edema by B-lines before and after the test.

**Results:**

Seventy-five patients were enrolled in the study, of whom 40% were in functional class IV on admission. The mean age was 58.3 ± 15.7 years and 40% of the patients were male. 95% patients accomplished the test and the average number of repetitions was 18 ± 7. No adverse event was recorded during or after the 1-min STST. Blood pressure, heart rate, and degree of dyspnea were increased after the test (all *p* < 0.001), while oxygen saturation was slightly decreased (97.0 ± 1.6 vs. 96.3 ± 2.0%, *p* = 0.003). The degree of pulmonary edema (χ^2^ = 8.300, *p* = 0.081) was not significantly changed, while there was a reduction in the absolute number of B-lines [9 (3, 16) vs. 7 (3, 13), *p* = 0.008].

**Conclusion:**

The application of the 1-min STST in early stage of ADHF appeared to be safe and feasible, which induce neither adverse event nor pulmonary edema. It may serve as a new tool of functional capacity assessment, as well as a reference of exercise rehabilitation.

## Introduction

Recently, a growing number of studies have found that most patients with acute decompensated heart failure (ADHF) suffer a severe reduction in functional capacity and quality of life due to the limitations in daily activities caused by HF and its complications (1). Severe physical impairment and frailty are major risk factors for adverse events in hospitalized elderly patients with ADHF (2–4). Exercise-based cardiac rehabilitation (CR) in patients with ADHF may change this vicious circle. The REHAB-HF study was the landmark clinical trial of CR in patients with ADHF. The transition of CR programs from inpatient to outpatient demonstrated that early, targeted, and progressive CR led to greater improvement in physical function than usual care (2). A retrospective study also found that early initiation of CR (within 2 days after admission) in ADHF was associated with shorter in-hospital stay, and lower 30-day HF readmission (1). However, exercise-based CR has not been routinely conducted in this group of patients due to the lack of accepted evaluation tools for functional capacity. The commonly used 6-min walking distance (6MWD) test and cardiopulmonary exercise test for chronic HF might not be applicable in early phase of ADHF when patients are almost confined to the ward because of serious conditions or weakness (5–7). Recently, the value of the 1-min sit-to-stand test (1-min STST) in pulmonary rehabilitation has drawn our attention, since it is convenient, space-saving, and easily monitored. The study by Reychler et al. confirmed that the 1-min STST could be an alternative to 6MWD test for exercise capacity in patients with chronic obstructive pulmonary disease (COPD) (8). It has also been validated in patients with cystic fibrosis (9) or COVID-19 (10). Undoubtedly, safety is a prerequisite before the 1-min STST can be considered as an assessment tool in patients with ADHF.

The degree of pulmonary edema is currently an important indicator of HF deterioration (11) and a direct semi-quantitative measurement of B-lines by Lung Ultrasound (LUS) has become an objective demonstration. By detecting the hyperechoic band artifacts with comet tails in the vertical direction (i.e., “comet-tail” signs or pulmonary edematous B-lines), LUS has been recommended for the diagnosis of acute respiratory distress of cardiac origin with high accuracy (12). It was reported that the sensitivity of LUS for acute pulmonary edema was 97% and the specificity was 98% (13). The number of B-lines not only correlates positively with the degree of pulmonary congestion, but also changes rapidly with the fluctuation of pulmonary circulation volume and pressure (14). Therefore, in the current study, we not only innovatively proposed the 1-min STST as the exercise test in patients with ADHF after the initial 48 h of admission, but also adopted the novel B-lines as the measures of pulmonary edema.

## Methods

### Study design

This was a prospective, single-center cohort study that conducted in the department of cardiology of a tertiary referral hospital (West China Hospital, Sichuan University) in China. Patients hospitalized with ADHF were screened for the eligibility. The diagnosis of ADHF complied with the current guidelines that was confirmed by a study cardiologist with expertise in HF (15). Additional inclusion criteria were (1) age ≥ 18 years; (2) ability to complete the sit-to-stand transitions on the chair without any external support; (3) normal cognitive function indicated by mini-mental state examination score > 16; and (4) ability to cooperate with the test. Exclusion criteria were (1) systemic diseases; (2) recent embolic event (<1 month) or thrombus in the heart; (3) acute coronary syndrome or recent coronary revascularization within 3 months; (4) acute endocarditis, myocarditis, pericarditis, or aortic dissection; (5) severe pulmonary hypertension, aortic stenosis, mitral stenosis, or hypertrophic obstructive cardiomyopathy; and (6) malignant arrhythmias or high-grade atrioventricular block. In accordance with the Declaration of Helsinki and the STROBE principle, the study protocol was approved by the Biomedical Ethics Committee of West China Hospital of Sichuan University (No. 20201276) and the registration at the China Clinical Trials Registry (ChiCTR2000040732) was completed.

### 1-min STST protocol

After the first 48 h of admission, eligible patients were screened by an experienced cardiologist and referred for initial assessment of the independent sit-to-stand transition, followed by conduction of the 1-min STST. The test was accomplished with the instruction and monitoring of a cardiac nurse and a physiotherapist, both of whom were blinded to baseline clinical data. It was performed on a standard-height chair with a seat height of 45 cm (16). The patients were requested to cross their arms and fold them across their chests and perform sit-to-stand transitions on the chair as many times as possible within 1 min. The test was started in a seated position with the patient’s back against the back of the chair (16). During the test, heart rate (HR) and oxygen saturation (SpO_2_) were monitored continuously by using pulse oximeter. Blood pressure (BP), degree of dyspnea by the Borg score, and the number of B-lines by LUS were measured before and after the test. The test would be interrupted if any adverse event was encountered (details as listed in Supplementary Table 1) (17–19), when the patient gave a sign to stop or the doctor considered it a high risk to continue. The major adverse events that recorded for analysis included chest pain, SpO_2_ < 85% for more than 1 min, presyncope or syncope, and patient-reported discomfort for discontinuation.

### Lung ultrasound

Lung ultrasound (LUS) examinations were performed before and after the 1-min STST by the trained cardiologist who also monitored the patients. The patients were asked to lie in a prone position and scanned by a hand-held echocardiography device (PHILIPS, Lumify S4-1, United States) with a phased-array transducer with an imaging depth of 18 cm in the sagittal direction. A 28-zone protocol was used for image acquisition as previously recommended (20). Briefly, anterolateral ultrasound scans of the chest were performed along the right and left hemithorax from the parasternal, midclavicular, anterior, and midaxillary lines of the second to fourth rib gaps (the fifth rib gap on the right). For each rib space, 3-s clips were captured and the number of B-lines was measured in real-time. The highest number of B-lines per zone was quantified throughout the segment and the sum of the 28 zones was used for analysis. A positive (+) was defined as the presence of >5 comets, otherwise a negative (−) pulmonary edema. The B-lines of 6–15, 16–30, and > 30 indicated mild, moderate, and severe pulmonary edema, respectively.

### Clinical congestion score

A clinical congestion score (CCS) was calculated for individual patient at baseline by the experienced cardiologist using a modified algorithm described by Kang et al. (21) (details as listed in Supplementary Table 2). A score of ≥3 was defined as CCS positive (+) and otherwise negative (−). The CCS of 3–6, 7–9, and ≥ 10 indicated mild, moderate, and severe congestion, respectively.

### Statistics analyses

Data were computed using IBM Statistical Package for Social Sciences (SPSS; version 25.0, New York, United States). Normally distributed continuous variables were presented as mean ± SD and non-normally distributed variables as median (interquartile ranges). Categorical variables were described by proportion and frequency count. The B-lines were analyzed as a continuous variable (the total number of B-lines) as well as a categorical variable (the four grades as abovementioned). The Paired Student’s *t*-test and Wilcoxon signed-rank test were used for the comparison before and after the 1-min STST. The Student’s *t*--test or Mann–Whitney U test was used when appropriate for the comparison between subgroups. Categorical variables were compared by χ^2^ test. Kendall’s tau correlation was used to assess the relationship between the change of B-lines and that of Borg score. A value of *p* < 0.05 (two-tailed) was considered statistically significant. G*Power statistical software (V.3.1；University of Dusseldorf, Dusseldorf, Germany) was used to conduct a *post hoc* power analysis based on Wilcoxon signed-rank test (matched pairs).

## Results

Seventy-five eligible patients (58.3 ± 15.7 years, 40% male) were enrolled in this study. Ninety-six patients were screened, but 20% of them were excluded mainly due to their inability to complete the sit-to-stand transitions by themselves. Baseline characteristics are presented in Table 1. All patients were ADHF who had a history of symptomatic HF, nearly 40% of them were in NYHA class IV on admission. By using CCS assessment, 34 (45.3%) patients had moderate or severe congestion, whereas 41 (54.7%) patients had negative or mild congestion. LUS demonstrated severe pulmonary edema in 6 (8.0%) patients, moderate in 16 (21.3%) patients, mild in 20 (26.7%) patients, and negative in 33 (44.0%) patients, respectively.

**Table 1:** Baseline characteristics.

Variables	Patients (*n* = 75)
Age, years	58.3 ± 15.7
Male, *n* (%)	30 (40)
BMI, kg/m^2^	23.8 ± 5.0
NYHA class, *n* (%)	
II	7 (9.3)
III	39 (52.0)
IV	29 (38.7)
Etiology, *n* (%)	
Ischemic cardiomyopathy	10 (13.3)
Dilated cardiomyopathy	30 (40.0)
Valvular heart disease	14 (18.7)
Others	11 (14.7)
Comorbidity, *n* (%)	
Atrial fibrillation	28 (37.3)
Anemia	27 (36.0)
Hypertension	29 (38.7)
Diabetes mellitus	15 (20.0)
Chronic obstructive pulmonary disease	7 (9.3)
LVEF, %	36.3 ± 14.5
≤ 40	51 (68.0)
> 40	24 (32.0)
LVEDD, mm	61.8 ± 11.4
E/e’	20 (14, 26)
eGFR, mL/min/m^2^	63 (40, 81)
NT-proBNP, ng/L	6,091 (3,411, 11,731)

Data presented as frequency (%) or mean ± SD or median (Quartile). eGRF, estimated glomerular filtration rate, mL/min/m^2^；LVEDD, left ventricular end-diastolic dimension; LVEF, left ventricular ejection fraction; NYHA, New York Heart Association; and NT-proBNP, N-terminal pro-B-type natriuretic peptide.

Seventy-one (95%) patients completed the 1-min STST, while the other four patients did not due to lower limb weakness. The average number of repetitions was 18 ± 7. There were four patients who exhibited a drop in SpO_2_ of more than 4% after the test, but not below 90%, and all returned to baseline level within 1 min. No major adverse event was recorded during or after the 1-min STST.

**Figure 1:**
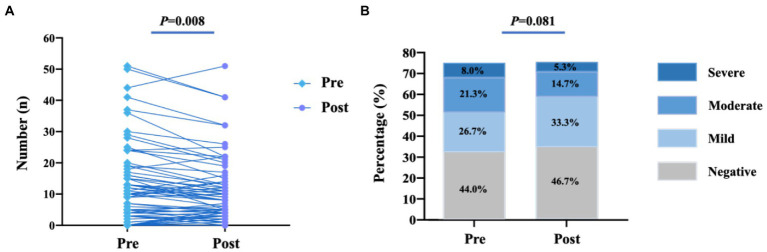
Changes of B-lines before and after the 1-min STST. **(A)** Number of B-lines; **(B)** Degree of pulmonary edema. There was significant changes in the numbers of B-lines [9 (3,16) vs. 7 (3,13), *p* = 0.008], while the degree of pulmonary edema (χ^2^ = 8.300, *p* = 0.081)was not significantly changed when compared with before the 1-min STST.

Table 2 lists the changes in vital signs and Borg score before and after the 1-min STST. The SBP, DBP, and HR increased after the test, while the SpO_2_ was slightly decreased. Although there was a significant increase in Borg score, only one patient reported a big change of six points. In addition, the number of B-lines decreased from 9 (3, 16) at baseline to 7 (3, 13) after the test (*p* = 0.008), and the absolute change in B-lines was −1 (−4, 1). By post-hoc power calculation, the power (1-beta) to detect this difference was 0.85 at an alpha coefficient of 0.05. However, the proportion of pulmonary edema by LUS (χ^2^ = 8.300, *p* = 0.081) was unchanged (Figures 1A,B). The number of B-lines was decreased or kept unchanged in 46 (61.3%) patients. It was increased in 29 (38.7%) patients, which was all less than five lines except six lines in one patient.

**Table 2:** Vital signs before and after 1-min STST.

	Pre 1-min STST	Post 1-min STST	*p* value
SBP, mmHg	104.6 ± 17.6	110.5 ± 19.8	<0.001
DBP, mmHg	69.0 ± 10.3	72.4 ± 10.9	<0.001
HR, bpm	85.1 ± 16.0	98.6 ± 17.6	<0.001
SpO_2_, %	97.0 ± 1.6	96.3 ± 2.0	0.003
Borg score	1.6 ± 1.2	3.8 ± 1.5	<0.001

Data presented as mean ± SD or median (Quartile).

DBP, diastolic blood pressure; HR, heart rate; SBP, systolic blood pressure; and SpO_2_, oxygen saturation.

**Figure 2:**
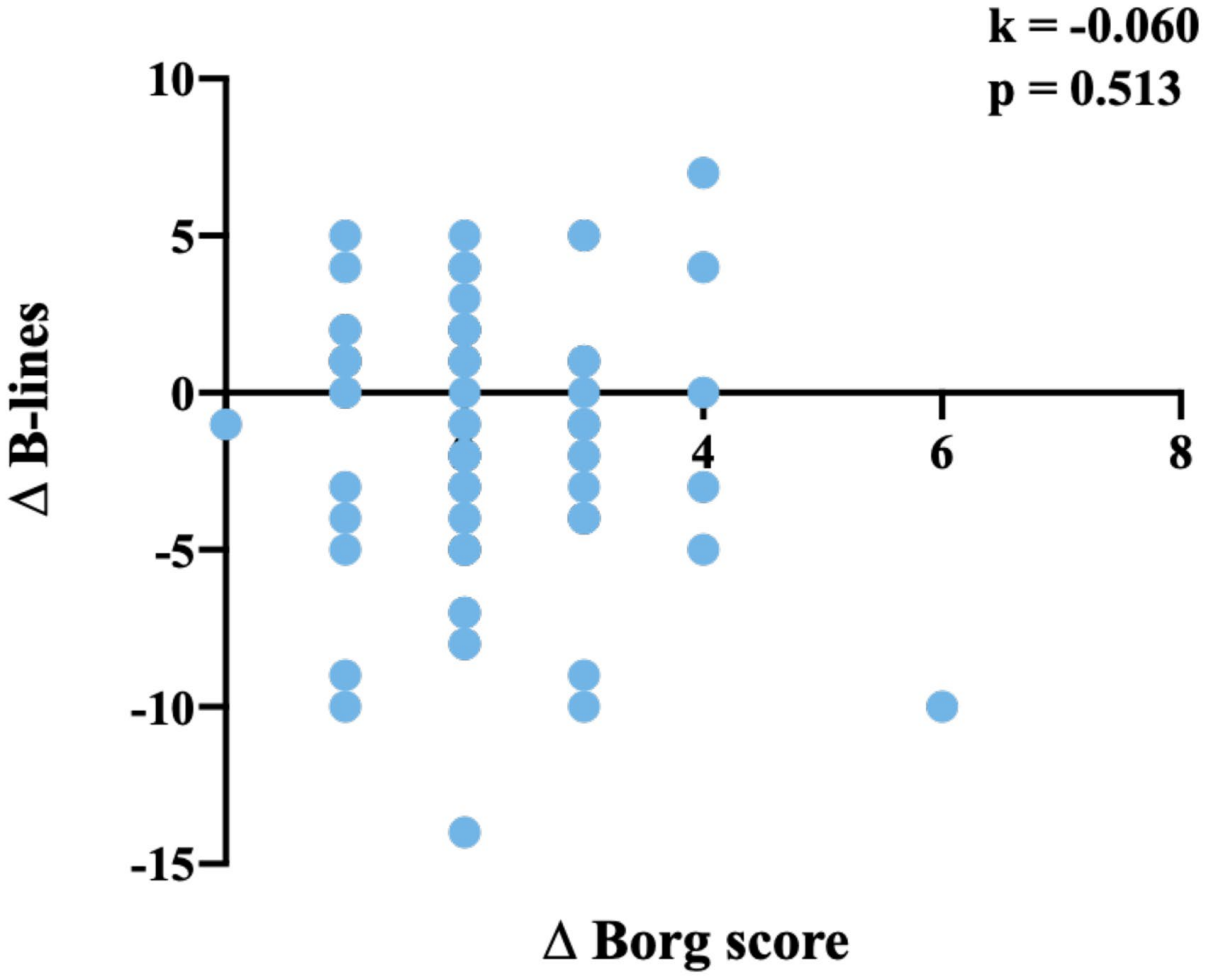
Correlation analysis of ∆B-lines and ∆Borg score. The scatterplot shows no correlation between the increase in the Borg score (∆Borg score) and the change in the number of B-lines (∆B-lines; *k* = −0.060, *p* = 0.513).

Patients were further divided into subgroups for comparison, including those CCS ≤6 vs. CCS >6, New York Heart Association (NYHA) classification II/III vs. IV, and left ventricular ejection fraction (LVEF) ≤ 40 vs, >40% (Table 3). Each subgroup showed a similar change in the vital signs after the 1-min STST, and there was not a difference between the defined two counterparts before and after the test. The Borg score was higher in the patients with CCS >6 than those with CCS ≤6 before and after the test, but the increase was not different between the two subgroups. The subgroup of CCS >6 was found to have more B-lines before and after the test than the other, as well as more significant reduction in the number. The comparison between the patients with NYHA class IV and those with NYHA class II/III showed a similar trend, while the difference between the patients with LVEF ≤40% and those with LVEF >40% was not that significant. The scatterplot shows no correlation between the increase in the Borg score (∆Borg score) and the change in the number of B-lines (∆B-lines; *k* = −0.060, *p* = 0.513; Figure 2).

**Table 3:** Subgroup comparison of vital signs and B-lines.

	CCS			NYHA class			LVEF (%)			≤ 6 (*n* = 41)	> 6 (*n* = 34)	*p* value	II/III (*n* = 46)	IV (*n* = 29)	*p* value	≤ 40 (*n* = 51)	> 40 (*n* = 24)	*p* value
Pre-SBP, mmHg	104.4 ± 16.7	104.9 ± 18.8	0.920	107.1 ± 18.5	100.7 ± 2.9	0.124	103.4 ± 17.8	107.3 ± 17.2	0.379
Post-SBP, mmHg	111.1 ± 19.5	109.8 ± 20.5	0.774	114.9 ± 19.6	104.5 ± 18.5	0.014	109.4 ± 19.2	112.8 ± 21.5	0.503
∆SBP, mmHg	9 (2, 13)	8 (0, 10)	0.468	10 (4, 13)	5 (−5, 10)	0.012	7 (1, 12)	9 (1, 12)	0.936
Pre-DBP, mmHg	69.9 ± 99	67.8 ± 10.9	0.378	69.9 ± 11.3	67.5 ± 8.5	0.329	68.8 ± 9.9	69.4 ± 11.3	0.795
Post-DBP, mmHg	73.2 ± 10.6	71.6 ± 11.4	0.535	74 ± 10.9	69.8 ± 10.7	0.117	72.8 ± 10.9	71.7 ± 11.2	0.699
∆DBP, mmHg	2 (−1, 8)	4 (1, 9)	0.373	3 (−1, 8)	3 (0, 9)	0.715	3 (−1, 10)	3 (0, 8)	0.477
Pre-HR, bpm	83.3 ± 14.3	87.2 ± 17.8	0.291	82.3 ± 14.3	89.0 ± 18.0	0.089	87.4 ± 17.0	80.1 ± 12.5	0.065
Post-HR, bpm	96.3 ± 17.6	101.3 ± 17.4	0.221	97.4 ± 17.4	100.4 ± 18.0	0.478	102.0 ± 17.2	91.3 ± 16.4	0.012
∆HR, bpm	12 (4, 20)	13 (7, 20)	0.583	14 (9, 21)	9 (4, 20)	0.112	13 (8, 20)	10 (3, 20)	0.169
Pre-SpO_2_, %	96.8 ± 1.6	97.2 ± 1.5	0.239	97.0 ± 1.5	96.9 ± 1.7	0.764	97.1 ± 1.6	96.8 ± 1.6	0.435
Post-SpO_2_, %	96.6 ± 2.1	96.0 ± 1.8	0.147	96.4 ± 1.7	96.3 ± 2.3	0.937	96.3 ± 1.9	96.5 ± 2.2	0.709
∆SpO_2_, %	0 (−1, 1)	−2 (−2.5, 0)	0.008	−1 (−2, 0)	0 (−2, 1)	0.703	−1 (−2, 0)	−1 (−2, 1)	0.518
Pre-Borg score	1 (0, 2)	3 (2, 3)	<0.001	1 (0, 2)	2 (1, 3)	0.010	2 (1, 3)	1 (0, 2)	0.186
Post-Borg score	3 (2, 4)	5 (4, 5)	<0.001	4 (2, 4)	5 (3, 6)	0.001	4 (3, 5)	3 (3, 4)	0.028
∆Borg score	2 (2, 3)	2 (2, 3)	0.811	2 (1, 2)	2 (2, 3)	0.043	2 (2, 3)	2 (1, 3)	0.532
Pre-B-lines	4 (0, 10)	15 (6, 24)	<0.001	5 (0, 13)	12 (3, 22)	0.033	10 (1, 18)	5 (3, 13)	0.561
Post-B-lines	4 (2, 11)	11 (5, 19)	0.002	5 (3, 11)	10 (4, 17)	0.134	8 (3, 13)	5 (3, 13)	0.657
∆B-lines	−1 (−2, 2)	−3 (−7, 0)	0.001	0 (−4, 2)	−2 (−5, 1)	0.094	−2 (−5, 1)	−1 (−2, 1)	0.307

Data presented as mean ± SD or median (Quartile). CCS, clinical congestion score; Post-, after the test; and Pre-, before the test. Other abbreviations as in Tables 1, 2.

## Discussion

We innovatively proposed the 1-min STST as a functional capacity assessment tool early stage of ADHF. As a result, 95% of patients accomplished the test with a mean number of repetitions of 18 ± 7. There was an increase of 3–6 mmHg in BP and 14 bpm in HR after the test, while the drop of SpO_2_ was within 1%. Despite a significantly higher Borg score of more than two points, the number of B-lines was not significantly increased after the 1-min STST.

Several studies have shown that the 1-min STST may be a good indicator of functional capacity. A systematic review that included 17 articles regarded the 1-min STST as a practical, reliable, valid, and responsive alternative method for measuring motor ability, especially in situations where space and time were limited (22). Pulmonary edema is one of the main features of HF that has been shown in numerous clinical trials to be associated with a significantly increased risk of death and rehospitalization (23). LUS with the measurement of B-lines is a tool for sensitive and reproducible semi-quantitative assessment of extravascular lung water, which provide a new way of real-time visualization of pulmonary edema. By using LUS, we demonstrated that the 1-min STST did not worsen pulmonary edema, though there was an increase in the degree of dyspnea by Borg score. It was attributed to a normal post-exercise reaction, like the increase in blood pressure and heart rate.

Therefore, the 1-min STST may provide a safe and feasible tool for the assessment of functional capacity in patients with ADHF that assist in establishing baseline and indication of exercise-based CR. At the same time, it may provide a common language among the members of multidisciplinary team, which may promote the exercise-based CR in this frail group of patients. In addition, the 1-min STST may also provide some reference values for creating risk prediction models of ADHF. It is no doubt that measures of functional ability (6MWD and PeakVO_2_) are important prognostic factors in HF patients (24, 25). A study by Gofus et al. found that the 1-min STST predicted the post-operative course of elective biological aortic valve replacement, in which the patients with a poorer pre-operative performance required a longer post-operative mechanical ventilation (26).

It is worth mentioning that patients with NYHA class IV on admission were also included in the current study. Traditionally, this group of patients was often excluded from CR due to the presence of dyspnea at rest and the fear of acute exacerbation in symptoms and vitals after exercise. However, in recent years, as research on CR in ADHF has gradually been intensified, patients in NYHA class IV have also started to be included in some studies (27). In this study, although patients in NYHA class IV had significantly more amounts of pulmonary water than patients in class II/III at baseline, they did not show an increase after the test.

The main strengths of this study were its novelty and the possibility of opening up a new field of research on functional capacity and early exercise-based CR in ADHF. However, there were some limitations. First, this was a single-center, small cohort study without substantial references for sample size estimation, though the *post hoc* power calculation was acceptable. Second, this study investigated on the safety of applying the 1-min STST in ADHF, but not further explored the threshold of starting CR or predicting outcome that was also very important. Third, with the focus on pulmonary edema and ability to accomplish the test, the study population may not be representative of all ADHF patients in our institution.

## Conclusion

The 1-min STST did not worsen HF symptoms or induce pulmonary edema in early stage of ADHF. It appeared to be a feasible and safe assessment tool for functional capacity in patients with ADHF, and perhaps a reference for exercise prescription in early CR.

## Data availability statement

The raw data supporting the conclusions of this article will be made available by the authors, without undue reservation.

## Ethics statement

The studies involving human participants were reviewed and approved by the Biomedical Ethics Committee of West China Hospital of Sichuan University (No. 20201276). The patients/participants provided their written informed consent to participate in this study.

## Author contributions

PY and QZ contributed to the conception and design of the work. XZ and YK contributed to the conduction of the study and preparation of the draft. ZL, QC, and MY contributed to the data collection and the critical revision of the manuscript. XZ and JZ contributed to the statistical analysis. All authors contributed to the article and approved the submitted version.

## Funding

This study was supported by the Major Project of the Science and Technology Department in Sichuan province China (grant number 2022YFS0112).

## Conflict of interest

The authors declare that the research was conducted in the absence of any commercial or financial relationships that could be construed as a potential conflict of interest.

## Publisher’s note

All claims expressed in this article are solely those of the authors and do not necessarily represent those of their affiliated organizations, or those of the publisher, the editors and the reviewers. Any product that may be evaluated in this article, or claim that may be made by its manufacturer, is not guaranteed or endorsed by the publisher.
