# Supplementary material for: Feasibility and safety of 1-min sit-to-stand test in acute decompensated heart failure confirmed by lung ultrasound
Source: Front Cardiovasc Med. 2023 Mar 8;10:1103247. doi: 10.3389/fcvm.2023.1103247 (PMC10030608; doi:10.3389/fcvm.2023.1103247)
Supplement: Supplementary file 1 [file Table_1.docx]

**Supplementary Table 1: ﻿Grading scale for CCS.**

| Signs/Symptoms | 0 | 1 | 2 | 3 |
| --- | --- | --- | --- | --- |
| NYHA class | I | II | III | IV |
| Borg score | 0 | 1-3 | 4-6 | 7-10 |
| Jugular venous distension | (-) |  | (+) |  |
| Rales | None | Bases | To < 50% | To > 50% |
| Peripheral oedema * | ﻿Absent | Mild | Moderate | Severe |

CCS = clinical congestion score; (-) = negative; (+) = positive; *: Mlid oedema is oedema below the ankle, moderate oedema is oedema below the knee, and Severe oedema is oedema above the knee

**Supplementary Table 2: Security Event Determination Criteria.**

| **Cardiovascular related events** | **Respiratory related events** | **Neurological related events** | **Other medical related Events** |
| --- | --- | --- | --- |
| - Angina/chest pain (ECG ischemic changes) - Severe cardiac arrhythmias (hemodynamic instability) - Cyanosis - Hypotension (MAP <60 mmHg/SBP <90 mmHg for more than 2 min) - Hypertension (MAP>140 mmHg/SBP>180 mmHg for more than 2 min) - Bradycardia (HR <50bpm and lasting more than 2 min) - Tachycardia (HR > 140 bpm for more than 2 min) - Cardiac arrest | - Dyspnea - Assisted respiratory muscle use - Reduced oxygen saturation: SpO_2_ <85% for more than 1min without oxygen; SpO_2_ <90% for more than 1min with oxygen - Excessive respiratory rate (RR > 30 bpm for more than 2 min). | - Syncope/loss of consciousness | - Adverse events considered by clinicians but not listed |

MAP = mean arterial pressure; SBP = systolic blood pressure; HR = heart rate; RR = respiratory rate
